# Young children’s use of probabilistic reliability and base-rates in decision-making

**DOI:** 10.1371/journal.pone.0268790

**Published:** 2022-05-25

**Authors:** Samantha Gualtieri, Elizabeth Attisano, Stephanie Denison

**Affiliations:** 1 Department of Psychology, University of Toronto, Toronto, Ontario, Canada; 2 Department of Psychology, University of Waterloo, Waterloo, Ontario, Canada; Dartmouth College, UNITED STATES

## Abstract

Children are skilled reasoners who readily use causal, reliability, and base-rate (i.e., prior probability) information in their decisions. Though these abilities are typically studied in isolation, children often must consider multiple pieces of information to make an informed decision. Four experiments (*N* = 320) explored the development of children’s ability to use reliability and base-rate information when making decisions about draw outcomes. Experiment 1 examined the age at which children can first compare and choose between probabilistically reliable machines. Three- and 4-year-old children saw machines that were probabilistically reliable at obtaining objects while sampling from uniform distributions (i.e., all target or non-target objects). Although 4-year-old children correctly used reliability in their decisions, 3-year-olds did not. In Experiment 2a, 4- to 6-year-olds were presented with the same probabilistically reliable machines, although they sampled from a mixture of target and non-target items. Here, children tended to choose the machine with the better proportion of targets, regardless of reliability. This was replicated in Experiment 2b. In Experiment 3, children were presented with one perfectly reliable machine and one probabilistically unreliable machine. Here, children continued to mostly choose the machine with the better proportion of targets. These results raise questions about *base-rate overuse* early in development and highlight the need for additional work on children’s ability to use multiple pieces of information in decision-making.

## Introduction

In order to maximize the value of our actions, we often have to consider multiple pieces of information in our decisions. For instance, imagine a child at a family picnic who would like to have some candy, but needs to be discrete because her parents have already said no. She knows that both her Aunt Mary and her Aunt Myra sometimes have candies in their purse, but she will likely only get away with covertly asking one before her parents figure out what she is up to. Perhaps she knows that Aunt Mary tends to have candy more often, but Aunt Myra is more likely to share if she happens to have some. So, who does the child ask? She could determine her odds of getting candy by prioritizing the probability of each aunt having candy, prioritizing the reliability of sharing for each aunt, or attempting to combine this information to come to a good intuitive estimate of her likelihood of receiving candy. In the current paper, we examine children’s ability to factor in base-rate and reliability information when making decisions about physical causal systems.

To navigate the uncertainty inherent in their environment, children need to make inferences using proportional information about a population, often referred to as base-rates. Infants, young children, and even some non-human animals compare numerical information across populations to determine which of two single-item samples is most likely to be of a target type [1–8]. In these paradigms, participants are presented with two populations that contain different numbers of target (preferred items or items associated with a reward) and non-target items. Each population contains a different proportion of targets and non-targets; for instance, Population A may contain 75% targets, while Population B may contain 25% targets. The experimenter randomly draws one occluded item from each population, placing them in separate locations, and the participant chooses a location to search in. Participants tend to choose the location with a sample from the population with more advantageous base-rates.

Children are also highly skilled causal reasoners [9–15]. That is, from very early in development, children can infer simple causal relations and structures after observing events in which some actions are reliably linked to outcomes and other actions are not. Most of this work has illustrated children’s proficiency when learning deterministic causal relations: If an object activates a machine all or none of the time (e.g., pressing the power button on your laptop turns it on, but pressing the volume button does not). However, some of the causal systems we interact with are *probabilistically* reliable (e.g., a coffee machine that works by a simple button press most, but not all, of the time). Although less work has examined probabilistic causes, young children can learn some causal relations from probabilistic evidence and use this information in their inferences and interventions [15–18]. In some of these experiments, children as young as 2 years old recognized that if a first action produces an outcome 67% of the time and a second action produces an outcome 33% of the time, they should intervene by using the first action because it is more efficacious [17, 18].

These independent literatures suggest that children use base-rate information to make decisions and that they can track cause-and-effect relationships, even when this information is probabilistic. However, much less is known about whether, and how, young children use this information *in tandem*. In the current experiments, we presented children with two causal machines to examine their use of base-rate and reliability information. To this end, the machines contained a base-rate of target and non-target objects, which were sampled using a claw-like mechanism. We varied the proportion of objects within each machine, along with the reliability of the claw at retrieving the objects. We asked children to choose between the machines for the best chance at obtaining a target object. We begin in Experiment 1 by establishing the age at which young children can make decisions that require a comparison between two probabilistically reliable causal machines, which, to our knowledge, has not yet been determined. Indeed, although 2-year-olds can track which of two actions activates a single machine most often when both actions are probabilistically effective [17, 18], we do not know whether children can track the reliability of two *separate* machines, compare across them to determine which is more reliable, and use that information to make a choice. Thus, because our task requires a more complex comparison across machines, we examine the emergence of this ability in 3- and 4-year-olds.

Following this, in three subsequent experiments, we examine whether children consider base-rate and reliability information in tandem in their decisions. There are a few possibilities: Children may begin by prioritizing one cue over the other. Base-rate information may be a particularly prominent cue for young children, considering that even infants use probabilistic information to inform their decisions [2, 3]. Moreover, recent evidence indicates that base-rate use may be a salient, intuitive response for young children when it is pitted against another piece of information [19, 20], consistent also with evidence suggesting that base-rate use is sometimes an intuitive response for adults [21]. Thus, children might prioritize base-rate information over reliability. Conversely, children may prioritize selecting the more reliable machine, regardless of base-rates, given their early sensitivity to causality [17, 18]. This prediction would also be consistent with the vast literature on base-rate neglect in adulthood [e.g., 22–24].

However, prior work also suggests that preschoolers and early school-aged children might consider both pieces of information in their decisions, and there may be developments in this ability throughout early childhood. For example, 4-year-olds consider numerical data in combination with a witness’ testimony [25, 26], 5-year-olds update their initial judgements based on numerical information in a task after being given additional evidence about identity [5], and 6-year-olds flexibly consider base-rates with personality information [20]. Furthermore, 5-year-old children have an emerging understanding of expected value, which requires integrating the likelihood of obtaining a reward with the value of that reward [27, 28]. That is, children adjust their judgments of how “good” a game is depending on the ratio of target to non-target items present, and the payout associated with the target item. Thus, after establishing the age at which children can first understand the probabilistic reliability information in this paradigm, the remainder of the experiments include children up to 6 years of age to capture any potential development in their ability to consider base-rate and reliability information in tandem.

In the current experiments, we presented children with problems in which the base-rate and reliability information conflicted. We were primarily interested in children’s use of one cue over the other (and, to what extent they varied their use of each cue across problems), and not if children could precisely compute the probabilities, per se, as these computations are very challenging. That is, when creating problems in which the cues conflict, the chances of each machine yielding a target can become quite close to one another. Thus, while children’s performance was coded for “correctness” (i.e., the machine that gives children the best chance of obtaining a target object, considering both base-rate and reliability), the main interest is in whether children’s responses are sensitive to shifting base-rates and reliabilities, rather than on whether they are systematically producing correct responses as a group on all trial types.

### Experimental approach

**Fig 1:**
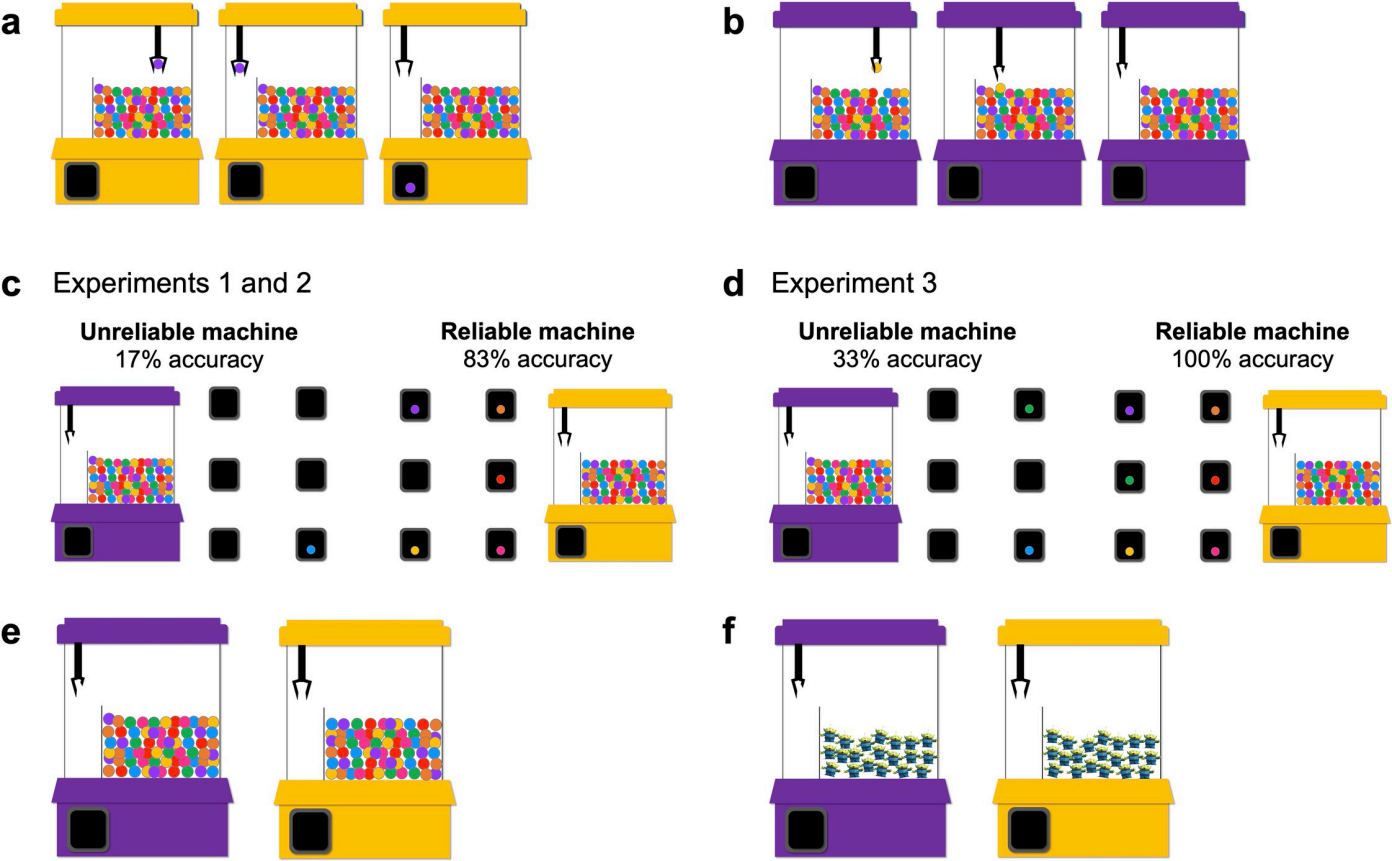
Overview of procedure. *Note*: Participants were introduced to two machines and were told that they were going to see how each machine worked before they played a game. Participants watched each machine succeed, or fail, at obtaining an item six times: The machine grabbed an object and successfully dropped it in the compartment (a), or the machine grabbed an object and dropped it in the container before it reached the compartment (b). Participants were then shown a recap of each machines’ reliability, depicted by the number of compartments that contained items (c and d). The reliability of each machine differed across Experiments 1 and 2 (c) and Experiment 3 (d). The experimenter reiterated whether the machine obtained an object on each try, for each of the compartments. Participants were then shown both machines together and were asked to indicate which was good at getting toys and which was not so good at getting toys (e). The experimenter agreed or disagreed with the participant’s answer and restated the reliability information. Participants then completed a set of test trials in which they chose between the two machines for the best chance at an alien toy (f). The test trials differed across experiments (see Methods).

Materials were presented on a laptop via PowerPoint and were narrated live by the experimenter (see Fig 1 for an overview). Participants were always introduced to two machines and were told that they were going to see how each worked before the game started. Note that although these machines somewhat resemble those seen at venues (i.e., where the container is filled with toys and a person manipulates a joystick that controls a claw to try to get a toy, thus, with some skill and agency involved), this was *not true* of the machines children saw in the experiments. That is, children saw evidence that each machine sampled items on its own and they knew that their actions were not controlling the machine. Children then saw a reliability sequence to establish the efficacy of each machine as they watched, sequentially, while the claw in each machine attempted to sample six items (see Fig 1A and 1B for examples of successful and unsuccessful attempts). The reliability of each machine differed across experiments (see Fig 1C for Experiments 1 and 2, and Fig 1D for Experiment 3). Children were then asked to indicate which machine was good at getting toys and which was not so good at getting toys (see Fig 1E). This was followed by a test phase, where children chose between machines to win a prize on different trial types (see Fig 1F for a sample problem from Experiment 1). The base-rate of targets to non-targets in each machine varied across trials. Trials in all experiments were named using the following convention: *number* Reliable *number* Unreliable, with *number* indicating the percent of targets in each machine for the trial (e.g., a problem labeled 10 Reliable 90 Unreliable indicates there were 10% targets in the reliable machine and 90% targets in the unreliable machine). Children’s responses were always coded for “correctness” and the correct response for every trial is bolded in the trial label. See S1 File for information on how the correct response was calculated for each problem. Data are available for all experiments, see “Data Availability” for details.

## Experiment 1

In Experiment 1, we asked 3- and 4-year-old children to choose between two probabilistically reliable causal machines to establish whether preschoolers can choose the more efficacious one. We presented children with two machines: One machine more reliably produced an outcome (obtained objects 83% of the time), and the other less reliably produced an outcome (obtained objects 17% of the time). The machines contained deterministic base-rates (i.e., either all target objects, or all non-target objects).

### Methods

#### Participants

This research received ethics clearance through the University of Waterloo Research Ethics Board, submitted under the name “Learning and conceptual development in infants and children” (protocol number: 30215). Informed written consent was obtained from guardians for all child participants. In all experiments, children were individually tested at schools, daycares, or museums in the Kitchener-Waterloo region or at a science centre in Toronto. Demographic information was not formally collected. The census indicates that approximately 81% of residents in Kitchener-Waterloo and 48.5% of residents in Toronto identify as White, and both the region and city are predominately middle-class [29, 30].

We tested 32 children per age in all experiments. This sample size was determined in advance based on the lab stopping rule at the time of data collection. Sixty-four children were included in the final analyses for Experiment 1, including 32 3-year-olds (*M*_*age*_ = 42.84 months, *female* = 15) and 32 4-year-olds (*M*_*age*_ = 53.27 months, *female* = 17). An additional three children were tested but excluded due to technical issues (*n* = 1), teacher interference (*n* = 1), and non-compliance (*n* = 1).

#### Experimental procedure

Participants were introduced to the two machines and were told that they were going to see how each machine worked before the game started (see Fig 1 for an overview). During the reliability sequence for Experiment 1, children watched, sequentially, as the claw in each machine attempted to sample six items. That is, children watched each claw succeed or fail at obtaining an item six times. The unreliable machine was able to successfully draw one item and missed five times (it retrieved an item on the last try; 17% accuracy), and the reliable machine was able to successfully draw five items and missed once (it retrieved an item on every try but the third try; 83% accuracy; see Fig 1C for an overview of the reliability). Note that both machines retrieved an item on the last try to ensure that children were basing their reliability judgments on the sequence of attempts, and not just the final trial. That is, if children were only paying attention to the last attempt in the sequence, they would believe that both machines were equally accurate at obtaining an item, because both machines drew an item on the final attempt (and thus would not pass the reliability question). Children then saw an overview of each machine’s reliability, and the experimenter summarized whether the machine retrieved an item or did not retrieve an item on each attempt. While viewing this slide, children were asked to indicate which machine was good at getting toys and which machine was not so good at getting toys (the reliability question). Though asked two separate questions, children (in all experiments) replied with one machine for the first question and the other machine for the second question, providing an answer that was consistently correct or incorrect. Thus, children received a score of 1 if they were correct on the reliability question. Depending on the child’s response, the experimenter agreed or disagreed with their answer and restated the reliability. This ensured that all participants, even those who answered incorrectly, were made aware of the overall reliability of both machines before moving on. After this, participants were told that they were going to play some games. To win the game, participants were told they needed to get alien toys and that they would see how many aliens they won at the end. Thus, there was no feedback on sampling for the test trials until the end.

**Fig 2:**
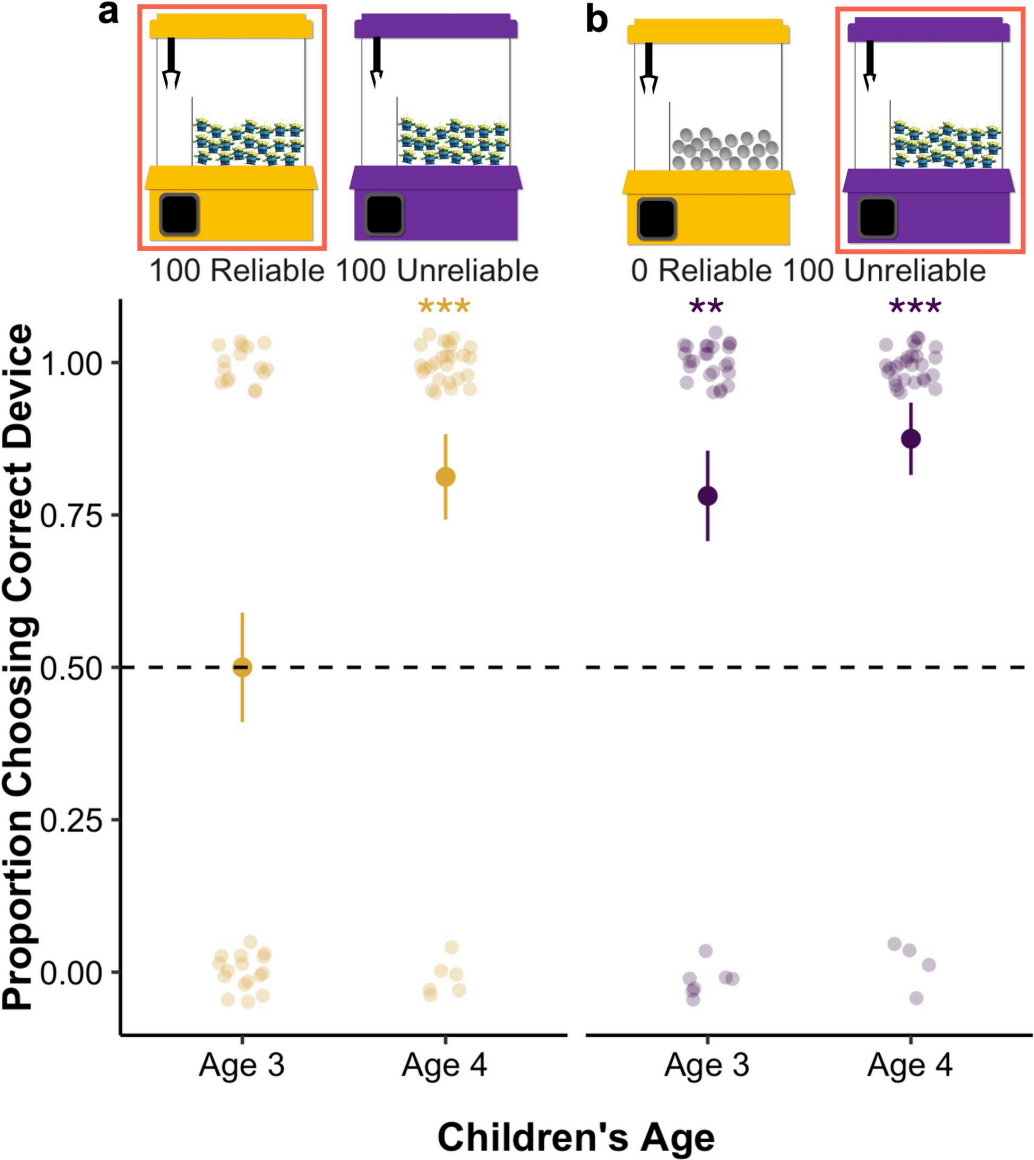
Overview of problems and children’s performance in Experiment 1. *Note*: *p ≤ .05, **p ≤ .01, ***p ≤ .001. The machine outlined in red is coded as correct for that problem. Mean performance and standard error bars are depicted, with individual data points jittered to avoid over plotting.

Children completed two problems (see Fig 2 for an overview of the problems). In the **100 Reliable** 100 Unreliable problem, both machines only contained alien toys. On this problem, children should choose the reliable machine, because it would give them a high chance of obtaining an alien toy, while the unreliable machine would give them a low chance (see S1 File for calculations for all experiments). This problem was diagnostic of children’s ability to choose between two machines that were probabilistically reliable. In the 0 Reliable **100 Unreliable** problem, the reliable machine contained 0% alien toys, while the unreliable machine contained 100% alien toys. On this problem, children should choose the unreliable machine, because it provides a chance at obtaining an alien toy, while the reliable machine contained no target items. This control problem is important because correct performance requires that children choose the unreliable machine. In absence of this problem, children’s performance on the **100 Reliable** 100 Unreliable problem would be difficult to interpret: A correct choice could mean that they can choose between two probabilistically reliable machines, or it could mean that they are unwilling to choose a machine labeled as “not very good”. On both problems, children received a score of 1 if they selected the optimal machine on each problem. In all experiments, the side of each machine (tied to reliability) and problem order were counterbalanced across participants.

### Results and discussion

First, we examined children’s responses to the reliability question using a logistic regression, including children’s age in months (mean centered) as a covariate. There was a significant effect of age on children’s responses to the reliability question, *Wald’s χ*^*2*^(*df* = 1) = 15.9, *p* < .001. For 3-year-olds, 22/32 children (69%) selected the correct machine, *p* = .05, binomial test. For 4-year-olds, 30/32 children (94%) selected the correct machine, *p* < .001, binomial test. Thus, children at these ages used the demonstrations to determine which machine is more reliable, although older children were notably better than younger children. Recall that the experimenter restated the reliability after the child answered this question, ensuring that even those who failed the reliability question were told which machine was more reliable before moving on to the test problems. Results of a logistic regression suggest that children’s performance on the reliability question did not affect their performance on the test problems, *Wald’s χ*^*2*^(*df* = 1) = .784, *p* = .36.

We then examined children’s responses to the test problems using a Generalized Estimating Equations (GEE) binary logistic regression that included problem (**100 Reliable** 100 Unreliable problem; 0 Reliable **100 Unreliable** problem) as a factor, children’s age in months (mean centered) as a covariate, and the interaction between problem and age in the model. This revealed a significant main effect of children’s age, *Wald’s χ*^*2*^(*df* = 1) = 11.74, *p* = .001. The effect of problem and the interaction between problem and age were not significant: problem, *Wald’s χ*^*2*^(*df* = 1) = 3.36, *p* = .07; problem x age, *Wald’s χ*^*2*^(*df* = 1) = .14, *p* = .71. See Fig 2 for an overview of children’s responses on each problem.

Because of the effect of age, we conducted follow-up binomial comparisons to chance, examining 3- and 4-year-old children’s performance separately. Three-year-olds chose the unreliable machine at rates higher than chance when it contained all target objects (0 Reliable **100 Unreliable** problem: *M* = .78, *SD* = .42, *p* = .002, binomial test), and performed at chance when both machines contained only target objects (**100 Reliable** 100 Unreliable problem: *M* = .50, *SD* = .51, *p* = 1, binomial test). This is particularly striking because the majority of 3-year-olds were correct when initially asked which machine was good at retrieving objects, though they did not consistently choose the reliable machine when base-rates were identical. Four-year-olds’ performance was significantly better than chance on both problems, as they chose the reliable machine when both machines only contained target objects (**100 Reliable** 100 Unreliable problem: *M* = .81, *SD* = .40, *p* < .001, binomial test), and the unreliable machine when it contained all targets (0 Reliable **100 Unreliable** problem: *M* = .88, *SD* = .34, *p* = .001, binomial test).

## Experiment 2a

In Experiment 1, 3- and 4-year-old children’s ability to track and choose between probabilistically reliable machines improved with age. To extend our examination, children were given more complicated problems in Experiment 2a, in which the base-rates were probabilistic (i.e., they contained a mixture of target and non-target objects). More specifically, we presented children with problems in which base-rates were pitted against the machine’s reliability, which, although quite difficult to solve, allowed for a test of any potential preferences or biases they may have when both pieces of information are presented in tandem. In light of discovering that 3-year-olds struggle with tracking and choosing between probabilistically reliable machines, we do not test 3-year-olds in subsequent experiments. Instead, we now include 5- and 6-year-old children, which allowed us to explore potential development in children’s use of the information in tandem, given previous work suggesting development in these abilities from 4- to 6-years of age [5, 20, 25–28].

### Methods

#### Participants

Ninety-six children were included in the final analyses, with 32 4-year-olds (*M*_*age*_ = 53.45 months, *female* = 17), 32 5-year-olds (*M*_*age*_ = 65.87 months, *female* = 12), and 32 6-year-olds (*M*_*age*_ = 78.52 months, *female* = 17) included in the final sample. An additional two children were tested but excluded due to non-compliance.

#### Experimental procedure

**Fig 3:**
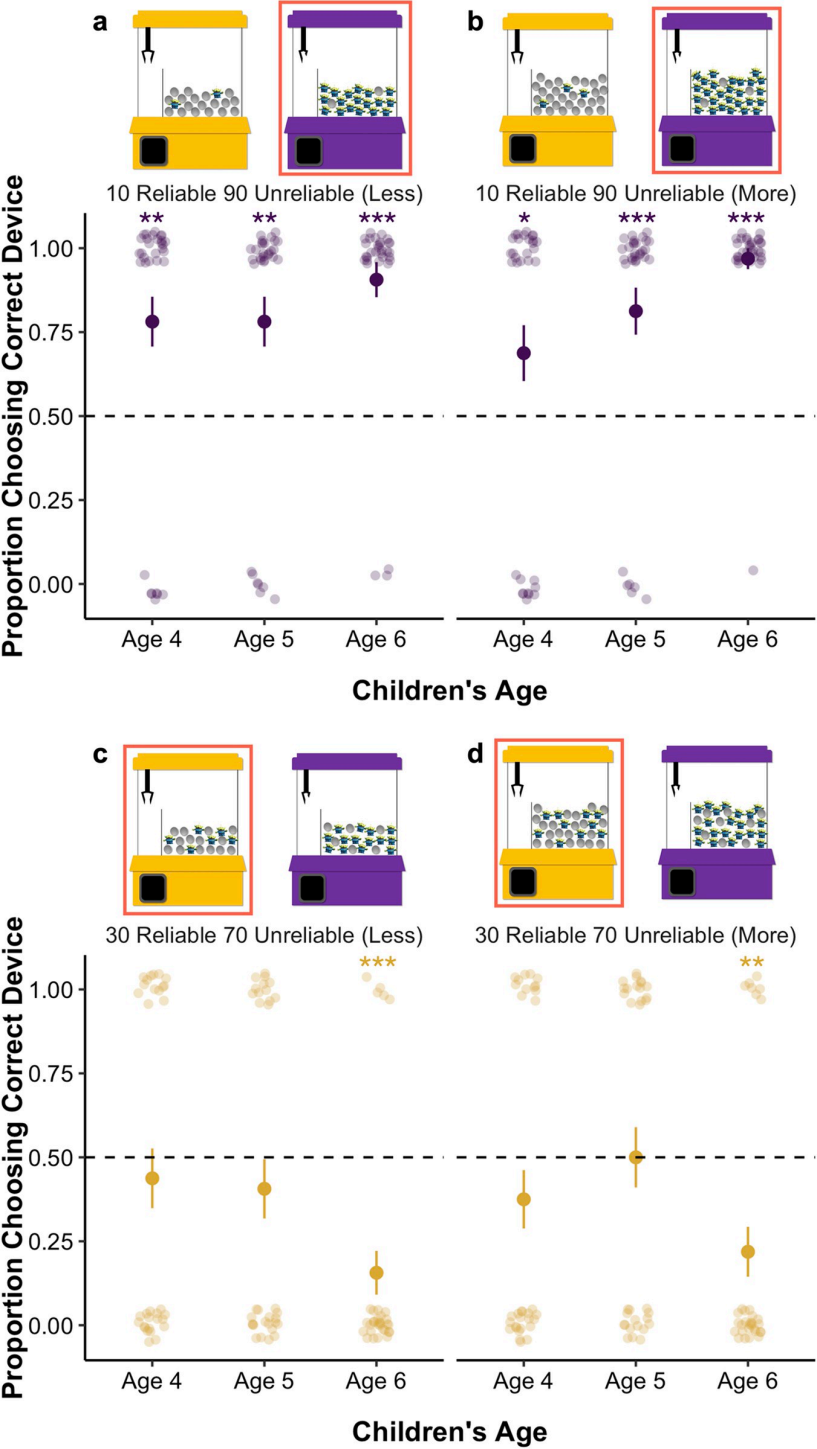
Overview of problems and children’s performance in Experiment 2a. *Note*: *p ≤ .05, **p ≤ .01, ***p ≤ .001. The machine outlined in red is coded as correct for that problem. Mean performance and standard error bars are depicted, with individual data points jittered to avoid over plotting.

The procedure was identical to Experiment 1, and the reliability of the machines remained the same (see Fig 1C for an overview of the reliability). However, children completed four problems that contained probabilistic base-rates (see Fig 3 for an overview of the problems). We created two problem types (i.e., 10 Reliable **90 Unreliable** and **30 Reliable** 70 Unreliable) and two versions of each (i.e., less and more). Problems of the same type contained the same proportions (e.g., both 10 Reliable **90 Unreliable** problems contained a comparison between 10% targets and 90% targets in the base-rates). Problem version determined the absolute number of objects in the population, in which problems labeled “more” contained a higher absolute number of objects. This allowed us to ask each child multiple questions of the same type without exact repetitions, and to explore if children’s use of base-rate information was affected by the absolute number of objects in the population. In 10 Reliable **90 Unreliable** problems, the reliable machine contained 10% alien toys, and the unreliable machine contained 90% alien toys. On these problems, the optimal choice is the unreliable machine, because it provides a better chance of obtaining an alien toy than the reliable machine. In **30 Reliable** 70 Unreliable problems, the reliable machine contained 30% alien toys, while the unreliable machine contained 70% alien toys. On these problems, the reliable machine is the optimal choice, because it provides a better chance of obtaining an alien toy than the unreliable machine. Thus, we pitted base-rates against the machine’s reliability by making the base-rates so extreme in the 10 Reliable **90 Unreliable** problems that the unreliable machine would be the better choice, and by making them less extreme in **30 Reliable** 70 Unreliable problems so that the reliable machine was the better choice. Children received a score of 1 if they selected the optimal machine on each problem.

### Results and discussion

As in Experiment 1, we first examined children’s responses to the reliability question using a logistic regression, including children’s age in months (mean centered) as a covariate. We did not find a significant age difference in children’s responses to the reliability question, *Wald’s χ*^*2*^(*df* = 1) = 2.24, *p* = .13. Overall, 95/96 children (99%) selected the correct machine, *p* < .001, binomial test. Children readily used the demonstrations to determine which machine was more reliable.

We then examined children’s responses to the test problems using a GEE binary logistic regression that included problem type (10 Reliable **90 Unreliable**; **30 Reliable** 70 Unreliable) and version (more, less) as factors, children’s age in months (mean centered) as a covariate, and all interactions. This revealed a significant main effect of problem type, *Wald’s χ*^*2*^(*df* = 1) = 38.40, *p* < .001; an interaction between problem type and age, *Wald’s χ*^*2*^(*df* = 1) = 7.47, *p* = .006, and an interaction between problem version and age, *Wald’s χ*^*2*^(*df* = 1) = 5.22, *p* = .02. All other main effects and interactions were not significant: problem version, *Wald’s χ*^*2*^(*df* = 1) = 1.23 *p* = .27; age, *Wald’s χ*^*2*^(*df* = 1) = 1.69, *p* = .19; problem type x problem version, *Wald’s χ*^*2*^(*df* = 1) = .03, *p* = .85; problem type x problem version x age, *Wald’s χ*^*2*^(*df* = 1) = 1.35, *p* = .25. See Fig 3 for an overview of children’s responses on each problem.

To explore the interactions, we conducted follow-up binomial tests comparing children’s performance separately at each age on each problem to chance (see Table 1 for means, standard deviations, and binomial tests). Recall that on these problems, reliability was pitted against base-rate information because the unreliable machine contained the better base-rate of target items. The base-rates were so extreme in the 10 Reliable **90 Unreliable** problems that the unreliable machine was the better choice, and were less extreme in **30 Reliable** 70 Unreliable problems such that the reliable machine was the better choice. On the 10 Reliable **90 Unreliable** problems, for both more and less versions, the performance of all age groups was significantly better than chance, with children selecting the unreliable machine with the better base-rate of target objects. On **30 Reliable** 70 Unreliable problems, for both versions, 4- and 5-year-old children’s performance was not significantly different from chance, suggesting that there was uncertainty around their choices as a group. However, 6-year-old children’s performance was significantly below chance on both versions, as they showed a clear preference for the base-rate and consistently chose the unreliable machine with the better base-rate of target objects, even though the reliable machine was the better choice.

**Table 1:** Children’s use of the correct machine on each problem in Experiment 2a.

	4-year-olds			5-year-olds			6-year-olds		
Problem	*M*	*SD*	*binomial p*	*M*	*SD*	*binomial p*	*M*	*SD*	*binomial p*
10 Reliable	.78	.42	.002	.78	.42	.002	.91	.30	< .001
**90 Unreliable** (Less)									
10 Reliable	.69	.47	.05	.81	.40	.001	.97	.18	< .001
**90 Unreliable** (More)									
**30 Reliable**	.44	.50	.60	.41	.50	.38	.16	.37	< .001
70 Unreliable (Less)									
**30 Reliable**	.38	.49	.22	.50	.51	1	.22	.42	.002
70 Unreliable (More)									

#### Individual strategy use

Because the base-rate and reliability information were pitted against each other in all four problems, we also coded children’s responses in terms of which machine they chose on each problem (i.e., the machine with the better base-rate or the more reliable machine), ignoring the item-type variable (more or less). This allowed us to classify children by their response patterns, which results in 9 unique patterns based on the combination of whether responses are correct and whether they correspond to a base-rate-favoring or reliability-favoring choice; they are fully described in Table 2. These response patterns are likely indicative of underlying strategies, such as favoring base-rates, favoring reliability, considering both pieces of information accurately, or responding randomly. Some response patterns might also be indicative of trying to employ a particular strategy but with some noise.

**Table 2:** Breakdown of children’s response patterns in Experiments 2a and 2b.

Response pattern	Description
**2 Base-rate 2 Reliable** (4 correct)^1^	Chose the correct response on all four trials (two choices that correspond to the better base-rate, two that correspond to the reliable machine).
**4 Base-rate** (2 correct)^1^	Chose the machine with the better base-rate on all four trials (two choices that correspond to a correct response, two that correspond to an incorrect response).
**3 Base-rate** (3 correct)^2^	Chose the machine with the better base-rate correctly on two trials and incorrectly on one trial. Chose the reliable machine correctly on one trial.
**3 Base-rate** (1 correct)^2^	Chose the machine with the better base-rate correctly on one trial and incorrectly on two trials. Chose the reliable machine incorrectly on one trial.
**4 Reliable** (2 correct)^1^	Chose the reliable machine on all four trials (two choices that correspond to a correct response, two that correspond to an incorrect response).
**3 Reliable** (3 correct)^2^	Chose the reliable machine correctly on two trials and incorrectly on one trial. Chose the machine with the better base-rate correctly on one trial.
**3 Reliable** (1 correct)^2^	Chose the reliable machine correctly on one trial and incorrectly on two trials. Chose the machine with the better base-rate incorrectly on one trial.
**2 Base-rate 2 Reliable** (2 correct)^3^	Chose the machine with the better base-rate correctly on one trial and incorrectly on one trial. Chose the reliable machine correctly on one trial and incorrectly on one trial.
**2 Base-rate 2 Reliable** (0 correct)^1^	Chose the incorrect response on all four trials (two choices that correspond to the better base-rate, two that correspond to the reliable machine).

Note

^1^1/16 potential response patterns

^2^2/16 potential response patterns

^3^4/16 potential response patterns.

**Fig 4:**
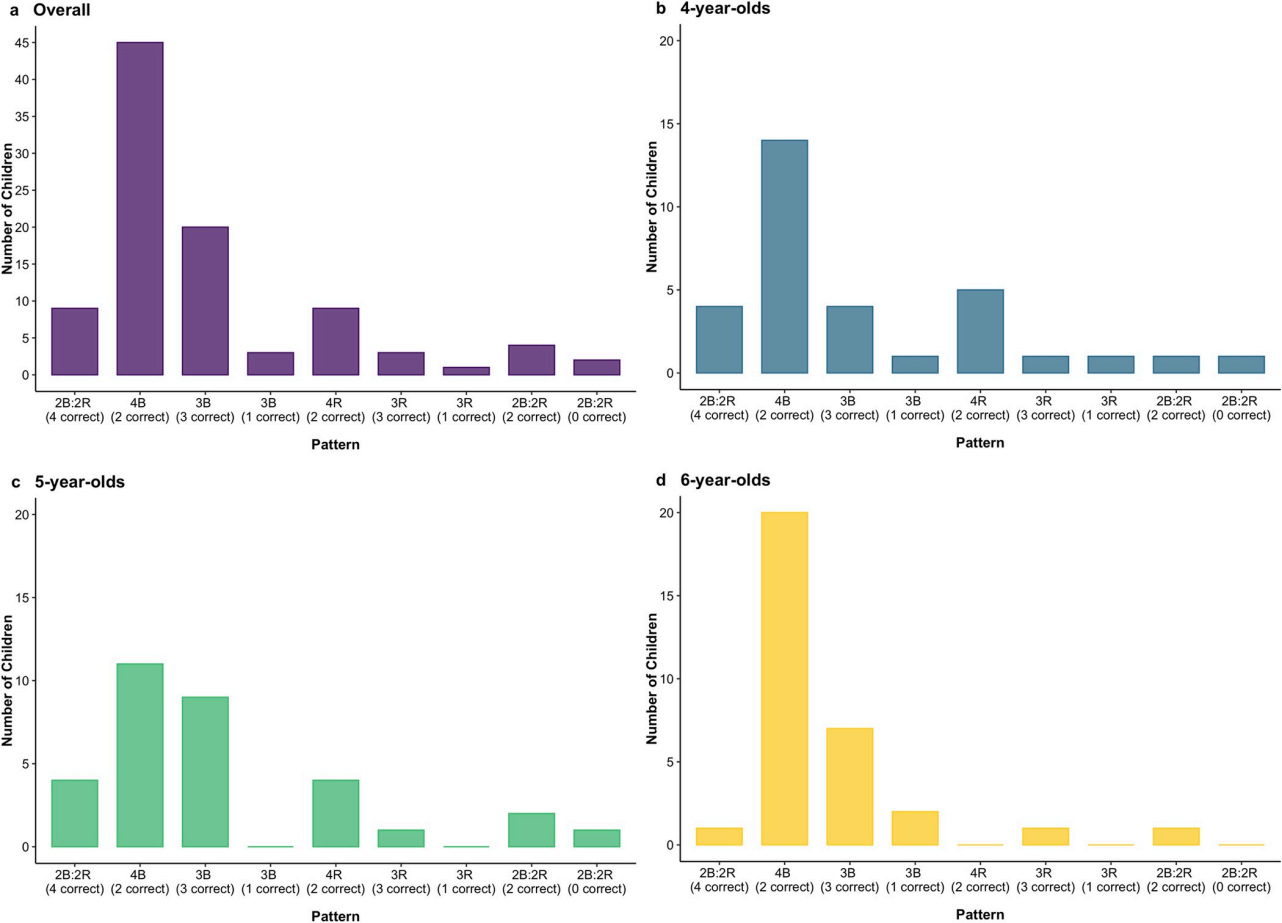
Children’s pattern of responses in Experiment 2a. *Note*: In the pattern names, “B” indicates selecting the machine with the better base-rate, “R” refers to selecting the reliable machine, and the number beside each letter indicates the number of times that machine was selected. See Table 2 for a breakdown of each pattern.

We classified children based on their response patterns and compared the proportion of children who produced such patterns to the proportion of children who would produce such a pattern by chance (see Table 3 and Fig 4). This should identify whether any response patterns are produced by children systematically and thus would be suggestive of a particular strategy being favored generally. Given that there are four problems, each with two potential responses, these can be thought of similarly to four coin flips, with 16 possible patterns and a 1/16 chance of producing each pattern at random. However, some patterns can occur in multiple ways (because problem types repeat) and these are identified in Table 2, and the chances of them occurring randomly are thus corrected to account for this. See Fig 4 and Table 3 for the number of children whose responses were characterized by each pattern, and the associated *p*-value for the comparison of the proportion of children producing the pattern and the proportion that would be expected to do so by chance. Note that because we computed 9 comparisons, *p*-values are significant if they are less than .0056. As is evident from Table 3, children most commonly fell into a base-rate preferring strategy, as the largest number of children (45/96) chose the machine with the better base-rate on every trial, and this was evident at all three ages (all *p’s* < .001, binomial tests). Another very common response pattern (20/96 children, not significantly above chance) was to get 3 of 4 questions correct via responding with the base-rate on 3 of 4 problems. This pattern is ambiguous, because it could indicate that children were trying to use both pieces of information accurately but erred on one problem, or that they were trying to always choose base-rates and erred on one problem. The other categories that diverged from chance were all significantly *lower* than chance values. Notably, very few children behaved entirely nonsensically, as only 2 of 96 children got every question wrong by choosing the machine with better base-rates when they should have chosen the more reliable machine and vice versa.

**Table 3:** Children’s pattern of performance in Experiments 2a and 2b.

	Experiment 2a								Experiment 2b	
	*Overall* (*N* = 96)		*4-year-olds* (*N* = 32)		*5-year-olds* (*N* = 32)		*6-year-olds* (*N* = 32)		*Overall* (*N* = 64)	
Pattern	*n*	*p*	*n*	*p*	*n*	*p*	*n*	*p*	*n*	*p*
**2 Base-rate**	9	.07	4	.09	4	.09	1	.27	3	.20
**2 Reliable** (4 correct)^1^										
**4 Base-rate** (2 correct)^1^	45	< .001*	14	< .001*	11	< .001*	20	< .001*	27	< .001*
**3 Base-rate** (3 correct)^2^	20	.007	4	.21	9	.009	7	.06	8	.15
**3 Base-rate** (1 correct)^2^	3	.001*	1	.06	0		2	.14	7	.15
**4 Reliable** (2 correct)^1^	9	.07	5	.03	4	.09	0		1	.07
**3 Reliable** (3 correct)^2^	3	.001*	1	.06	1	.06	1	.06	3	.02
**3 Reliable** (1 correct)^2^	1	< .001*	1	.06	0		0		1	.002*
**2 Base-rate 2 Reliable** (2 correct)^3^	4	< .001*	1	.001*	2	.006	1	.001*	12	.06
**2 Base-rate 2 Reliable** (0 correct)^1^	2	.04	1	.27	1	.27	0		2	.14

Note

^1^1/16 potential response patterns, compared to chance value of .0625

^2^2/16 potential response patterns, compared to chance value of .125

^3^4/16 potential response patterns, compared to chance value of .25. *p*-value with asterisks indicate a departure from chance based on the correct *p*-value (for multiple tests) of .0056.

## Experiment 2b

In Experiment 2a, we presented 4- to 6-year-olds with two machines that were probabilistically reliable and contained a mixture of target and non-target items and found that children generally prioritized base-rate information, even on problems where they should have chosen the reliable machine. This pull toward base-rate information was evident when we examined responses at the group level and when we examined individual strategies across problems. This base-rate overreliance was particularly pronounced in 6-year-olds, suggesting that there might be age-related differences in how children reconcile this information. However, one possibility is that the design of our paradigm contributed to base-rate over reliance. That is, the base-rate information was readily available to children when they made their decision on each test trial because the populations were in full view when they chose between the two machines. The reliability information, on the other hand, was kept in memory, with the slightly misshapen appearance of the unreliable claw serving as a reminder. To address the possibility that the presence of base-rates and relative absence of reliability information caused base-rate over reliance, we ran a follow-up experiment with 5- and 6-year-olds. This procedure was identical to Experiment 2a, except we hid the contents of the machine while children were making their decisions.

### Methods

#### Participants

Sixty-four children were included in the final analyses, with 32 5-year-olds (*M*_*age*_ = 66.76 months, *female* = 16) and 32 6-year-olds (*M*_*age*_ = 77.33 months, *female* = 16). An additional child was tested and excluded due to experimenter error.

#### Experimental procedure

The procedure was identical to Experiment 2a; however, the contents (i.e., base-rates) of the machines were covered before children made their choices.

### Results and discussion

First, we examined children’s responses to the reliability question using a logistic regression, including children’s age in months (mean centered) as a covariate. We did not find a significant age difference in children’s responses to the reliability question, *Wald’s χ*^*2*^(*df* = 1) = 1.04, *p* = .31. Overall, 62/64 children (97%) selected the correct machine, *p* < .001, binomial test.

**Fig 5:**
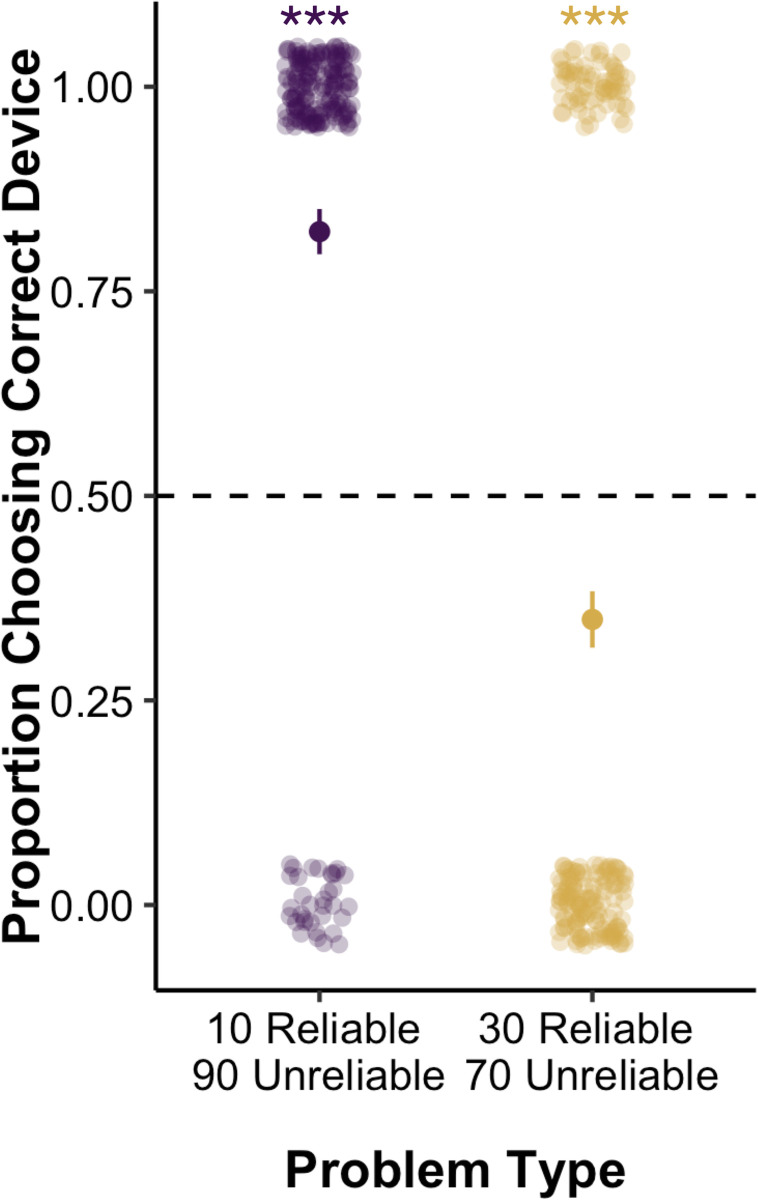
Children’s performance in Experiment 2b. *Note*: *p ≤ .05, **p ≤ .01, ***p ≤ .001. Mean performance and standard error bars are depicted, with individual data points jittered to avoid over plotting.

We then examined children’s responses to the test problems, with coding and analyses identical to Experiment 2a. There was a significant main effect of problem type, *Wald’s χ*^*2*^(*df* = 1) = 38.68, *p* < .001. All other main effects and interactions were not significant: problem version, *Wald’s χ*^*2*^(*df* = 1) = 1.13 *p* = .29; age, *Wald’s χ*^*2*^(*df* = 1) = .22, *p* = .64; problem type x problem version, *Wald’s χ*^*2*^(*df* = 1) = 2.20, *p* = .14; problem type x age, *Wald’s χ*^*2*^(*df* = 1) = .18, *p* = .67; problem version x age, *Wald’s χ*^*2*^(*df* = 1) = 1.91, *p* = .17; problem type x problem version x age, *Wald’s χ*^*2*^(*df* = 1) = 2.84, *p* = .09. See Fig 5 for an overview of children’s responses on each problem.

We conducted follow-up binomial tests comparing children’s performance on each problem to chance. Recall that on these problems, reliability was pitted against base-rate information because the unreliable machine contained the better base-rate of target items. On 10 Reliable **90 Unreliable** problems, children’s performance was significantly better than chance as they selected the unreliable machine with the better base-rate of target objects (*M* = .77, *SD* = .43, *p* < .001, binomial test). On **30 Reliable** 70 Unreliable problems, children’s performance was significantly worse than chance as they selected the unreliable machine with the better base-rate of target objects (*M* = .27, *SD* = .45, *p* < .001, binomial test). Thus, children showed a preference for the machine with the better proportion of objects, even though the population was covered while they made their choice.

#### Individual strategy use

**Fig 6:**
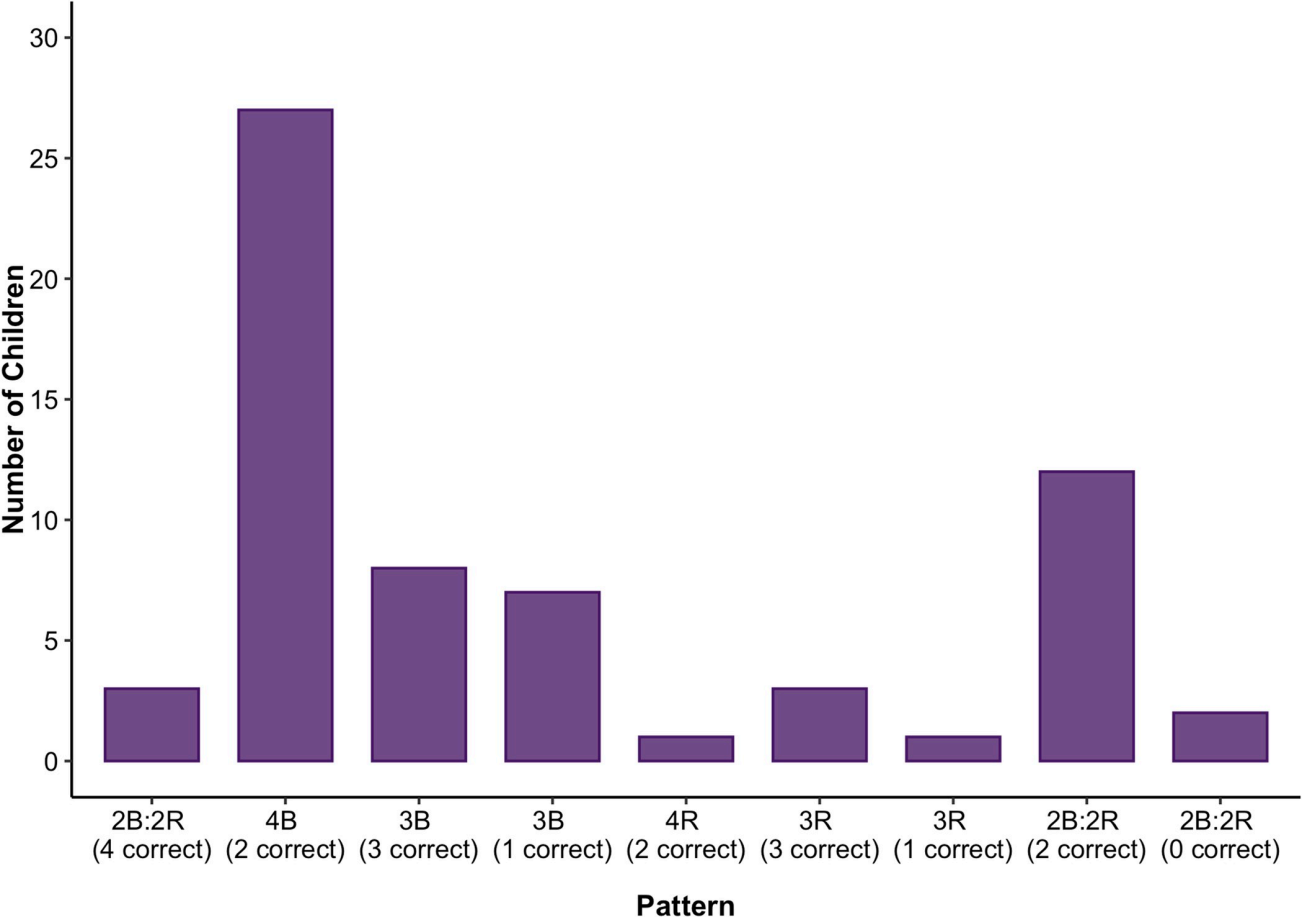
Children’s pattern of responses in Experiment 2b. *Note*: In the pattern names, “B” indicates selecting the machine with the better base-rate, “R” refers to selecting the reliable machine, and the number beside each letter indicates the number of times that machine was selected. See Table 2 for a breakdown of each pattern.

As in Experiment 2a, we examined children’s strategy use across problems (see Table 2 for a breakdown of each pattern) and compared the proportion of children classified into each pattern to the proportion of children that would be expected to fall into that pattern by chance. See Fig 6 and Table 3 for the number of children whose responses were characterized by each pattern, and the associated *p*-values, corrected for multiple tests. Even with the base-rates hidden at the time of their choice, children (27/64) tended to choose the machine with the better base-rate on all problems (*p* < .001, binomial test). Again, there was one strategy that was significantly different from chance because it occurred at *below* chance levels.

In sum, children in Experiment 2b continued to prioritize the base-rate information, even though the base-rates were hidden at the time of their decision, suggesting that in this paradigm their preference for base-rates is rather persistent. Similar manipulations have weakened children’s base-rate preferences in other tasks [31]; that is, hiding base-rates has made children more likely to integrate alternative information with base-rates, but that was not found here. Additionally, there is much evidence in favor of a “description-experience” gap in probability paradigms [32], in that probability information is often better encoded and used when experienced sequentially (as in our reliability sequence), rather than described summatively (as in our presented base-rates). Thus, if anything, we might have expected greater reliance on the reliability information in this paradigm. Given all this, children’s continued overreliance on base-rates in this modified paradigm is surprising.

## Experiment 3

In Experiment 3, we try one additional manipulation to further explore the extent of children’s apparent base-rate overreliance. To this end, we manipulated the reliability of the machines by presenting children with one machine that was probabilistically unreliable (obtained objects 33% of the time) and one that was *always* reliable (obtained objects 100% of the time). Providing children with a machine that is always reliable might make it easier for them to factor in the reliability information with the base-rate. In other work on causal reasoning, making at least one activation pattern deterministic has improved children’s performance [15]. Because the machine is always accurate, choosing the unreliable machine with a better base-rate here would suggest a very strong preference for base-rate information early in development. This change also allows us to construct problems in which the probabilities between the two machines are further apart than those in Experiment 2, making the problems easier to solve mathematically.

### Methods

#### Participants

Ninety-six children were included in the final analyses, with 32 4-year-olds (*M*_*age*_ = 53.69 months, *female* = 15), 32 5-year-olds (*M*_*age*_ = 64.74 months, *female* = 20), and 32 6-year-olds (*M*_*age*_ = 77 months, *female* = 15) included in the final sample. An additional three children were tested but excluded due to sibling interference (*n* = 1), and non-compliance (*n* = 2).

#### Experimental procedure

The procedure was identical to Experiments 1 and 2, though we changed the reliability of the machines (see Fig 1D for an overview of the reliability). The unreliable claw was able to successfully draw two balls (33% accuracy), and the reliable machine was able to successfully draw six balls (100% accuracy).

**Fig 7:**
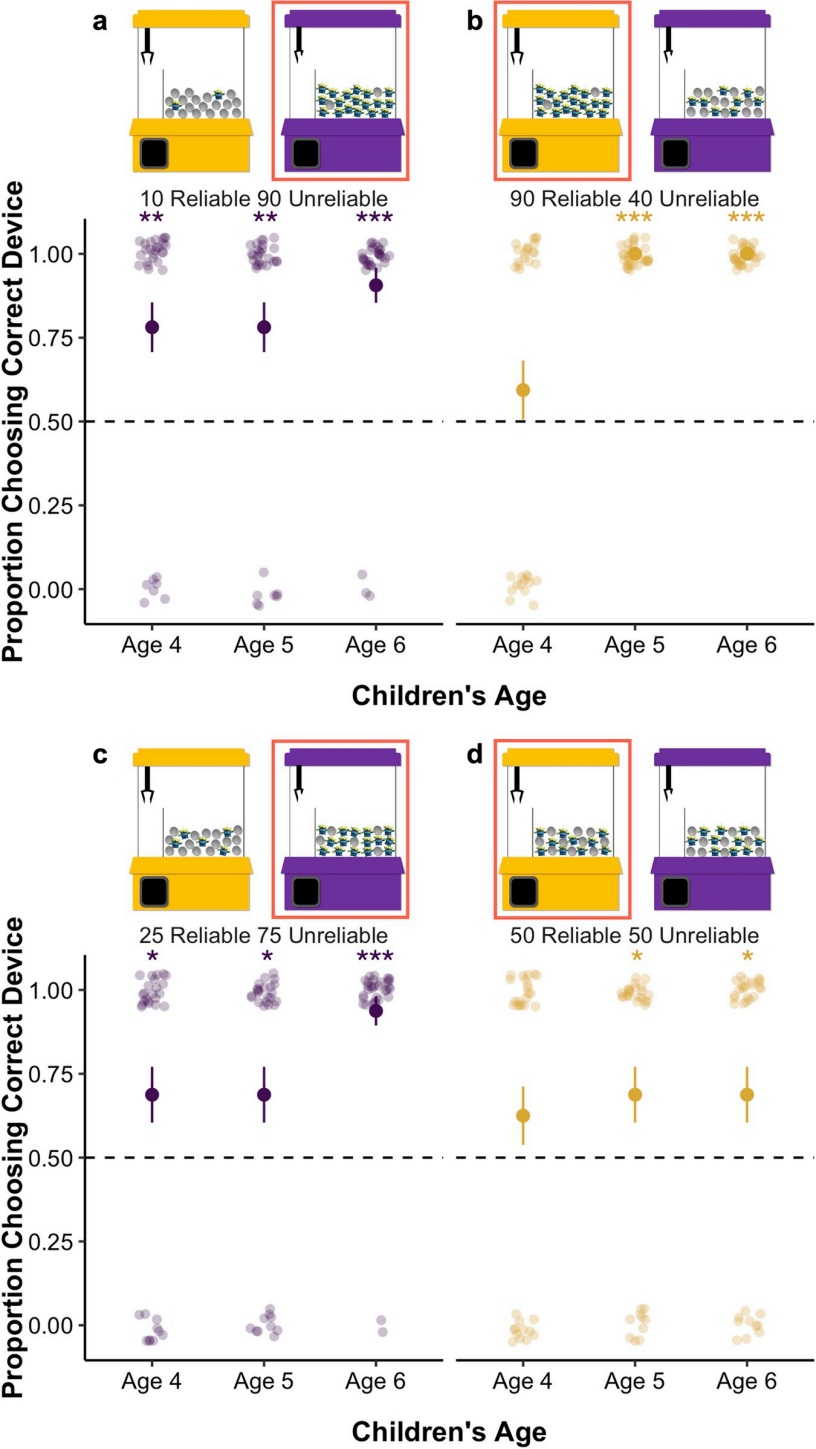
Overview of problems and children’s performance in Experiment 3. *Note*: *p ≤ .05, **p ≤ .01, ***p ≤ .001. The machine outlined in red is coded as correct for that problem. Mean performance and standard error bars are depicted, with individual data points jittered to avoid over plotting.

Participants completed four problems (see Fig 7 for an overview of the problems). In the 10 Reliable **90 Unreliable** problem, the reliable machine contained 10% alien toys, and the unreliable machine contained 90% alien toys. On this problem, the unreliable machine is the optimal choice, because it provides a better chance of obtaining an alien toy than the reliable machine. In the **90 Reliable** 40 Unreliable problem, the reliable machine contained 90% alien toys, while the unreliable machine contained 40% alien toys. On this problem, the reliable machine is the optimal choice, because it provides a better chance of obtaining an alien toy than the unreliable machine. In the 25 Reliable **75 Unreliable** problem, the reliable machine contained 25% alien toys, and the unreliable machine contained 75% alien toys. Here, both machines gave children approximately the same chance of obtaining an alien toy, but the unreliable machine had a better base-rate of target items. This problem is interesting because children should choose at chance, and thus any consistent tendency toward one machine would provide evidence for a preference for that piece of information. In the **50 Reliable** 50 Unreliable problem, both machines contained 50% alien toys. On this problem, the reliable machine is the optimal choice. This problem is also particularly diagnostic; performance around chance would suggest that children are focusing on the base-rate information without considering the reliability of the machines in their decision. Children received a score of 1 if they selected the optimal machine on each problem. Because there was no optimal choice for the 25 Reliable **75 Unreliable** problem (as both machines yielded approximately the same expected value), children received a score of 1 if they selected the unreliable machine, which was consistent with the base-rate. Because of how these problems were constructed, we were unable to examine individual strategy use across problems as we did in Experiments 2a and 2b: The base-rate and reliability information aligned in **90 Reliable** 40 Unreliable problem, and strategy use in the **50 Reliable** 50 Unreliable problem was difficult to classify.

### Results and discussion

First, we examined children’s responses to the reliability question using a logistic regression, including children’s age in months (mean centered) as a covariate. We did not find a significant age difference in children’s responses to the reliability question, *Wald’s χ*^*2*^(*df* = 1) = 0.04, *p* = .84. Overall, 93/96 children (97%) selected the correct machine, *p* < .001, binomial test.

We then examined children’s responses to the test problems using a GEE binary logistic regression that included problem (10 Reliable **90 Unreliable**; **90 Reliable** 40 Unreliable; 25 Reliable **75 Unreliable**; **50 Reliable** 50 Unreliable) as a factor, children’s age in months (mean centered) as a covariate, and the interaction between problem and age in the model. This revealed a significant main effect of problem, *Wald’s χ*^*2*^(*df* = 3) = 23.25, *p* < .001, age, *Wald’s χ*^*2*^(*df* = 1) = 29.34, *p* < .001, and an interaction between problem and age, *Wald’s χ*^*2*^(*df* = 3) = 19.36, *p* < .001. See Fig 7 for an overview of children’s performance on each problem.

To further explore the interaction between problem and age, we conducted follow-up binomial tests comparing children’s performance separately on each problem to chance (see Table 4). At all ages, children’s responses were significantly better than chance on the 10 Reliable **90 Unreliable** problem, as they opted for the unreliable machine with the better base-rate of target items. Five- and six-year-olds performed at ceiling on the **90 Reliable** 40 Unreliable problem, selecting the perfectly reliable machine that also contained the better base-rate. The responses of 4-year-olds, however, did not differ from chance on this problem. Across the age groups, children’s responses were significantly different from chance on the 25 Reliable **75 Unreliable** problem. Recall that on this problem both machines yielded approximately the same expected value, so children’s systematic preference for the unreliable machine indicates that they are relying on the base-rate in their decision. On the **50 Reliable** 50 Unreliable problem, 5- and 6-year-olds selected the reliable machine at rates above chance, which was the optimal decision, while 4-year-olds’ performance did not differ from chance. Because both machines contained the same base-rate on this problem, 5- and 6-year-olds’ performance indicates that they are considering the reliability information in their inferences when multiple pieces of information are relevant to their decision, but the base-rates are neutral (i.e., 50%). Thus, across these problems, children tend to be pulled by base-rate information when it conflicts with reliability, suggesting a strong preference for base-rates early in development.

**Table 4:** Children’s use of the correct machine on each problem in Experiment 3.

	4-year-olds			5-year-olds			6-year-olds		
Problem	*M*	*SD*	*binomial p*	*M*	*SD*	*binomial p*	*M*	*SD*	*binomial p*
10 Reliable	.78	.42	.002	.78	.42	.002	.91	.30	< .001
**90 Unreliable**									
**90 Reliable**	.59	.50	.38	1	0	< .001	1	0	< .001
10 Unreliable									
25 Reliable	.69	.47	.05	.69	.47	.05	.94	.25	< .001
**75 Unreliable**									
**50 Reliable**	.63	.49	.22	.69	.47	.05	.69	.47	.05
50 Unreliable									

## General discussion

In four experiments, we examined 3- to 6-year-old children’s ability to consider base-rate and reliability information in their decisions. In Experiment 1, we found that by age 4, children use probabilistic reliability to make decisions across two machines. This is particularly striking because there was a disconnect in 3-year-olds’ ability to use the reliability information in their decisions; the majority of 3-year-olds correctly stated which machine was more reliable, but they struggled to apply this information when making a concrete choice between machines. In Experiment 2a, we found that 4- to 6-year-old children had a general preference for the machine with a better base-rate of target items and were less sensitive to the machine’s reliability as most children opted to employ a base-rate strategy across problems. In Experiment 2b, 5- and 6-year-olds continued to prioritize base-rate information when the base-rates were hidden during the time of their choice, indicating that this preference is not due to a potential confound in our design. In Experiment 3, we explored the extent of this preference with a perfectly reliable machine. Five- and six-year-old children still chose the machine with the better base-rate of target objects, though they appropriately opted for the reliable machine when the base-rates were equivalent, showing that they can use the reliability when base-rates are uninformative. Four-year-olds’ ability to reconcile this information was fragile: Although they generally chose the machine with the better base-rate of target items, they were much more inconsistent. Together, these experiments suggest that there is development in children’s use of reliability and base-rate information in early childhood.

The current findings lend support to the argument that base-rate use may be a prepotent, intuitive response for young children in some contexts. Non-human primates, monkeys, and even parrots rely on base-rate information to inform their choice behavior, indicating that probabilistic inference is an evolutionarily ancient ability [1, 4, 7, 8]. Indeed, infants use probabilistic information to predict events and inform their choices, suggesting that probabilistic intuitions emerge early in human development [2, 3, 33, 34]. Given this early ability, children may accumulate experience using base-rate information, making it relatively easy and perhaps even automatic for them to use base-rate information in their decisions. This appearance of probabilistic intuitions in human infants and non-human animals adds plausibility to the notion of a strong predisposition toward base-rate use in simple, visual decision-making tasks [35]. This may appear to contrast starkly with adults, who frequently discredit or ignore the base-rates in lieu of personality information [22–24]. However, we do not wish to make strong claims or connections between the adult base-rate neglect literature and the present findings. This task is different from problems that reveal base-rate neglect in adults in numerous important ways, including that the competing information in adult problems is often a piece of information that aligns closely with a heuristic response based on social stereotypes or other well-practiced information. Here, base-rates are pitted against probabilistic physical causal information that would not align with any heuristic responses.

Children’s preference for base-rate information might also arise from misconceptions about their ability to win. Indeed, prior work has shown that children favorably predict their own performance in general [36, 37]. Further, younger children are more prone to optimism biases in games of chance than are their older counterparts. When making predictions, 7- to 10-year-olds provide estimates in line with the likelihood of an outcome, while 3- to 6-year-olds are more likely to base their predictions on the attractiveness of the reward associated with the outcome [38]. Thus, in the current experiments, young children’s limited appreciation for the machines’ reliability may be due in part to optimism regarding their ability to win a desired object on the single draw. Put another way, children might see the distribution of prizes and simply be drawn to the machines with more of those prizes due to wishful thinking that the single draw for them will result in a target item being sampled, regardless of the machine’s reliability. One problem with this interpretation is that if children were simply being optimistic, it is difficult to know why the base-rate proportions would factor so heavily into their reasoning. If children were optimistic and simply thought they would always get the item they wanted, then the base-rate would also be irrelevant (unless it contained no targets), as children should assume that they would sample the desired item no matter how improbable it was based on the proportions. It is possible that an optimism bias contributed to 3-year-olds’ performance in Experiment 1, as they selected the correct machine when only one machine contained target objects and performed at chance when both machines contained target objects. However, because this pattern of performance could also result from an issue tracking the machines’ reliability, we cannot make strong claims about 3-year-olds’ behavior. Future work is needed to examine how optimism biases impact children’s use of information when making decisions about sampling outcomes.

That said, children’s limited use of the machine’s reliability is somewhat surprising given their early sensitivity to the effectiveness of causal actions [17, 18], and given their ability to use reliability information in other paradigms [25, 26]. One possibility is that children struggled making inferences based on our novel machines because they were unfamiliar with the mechanism used in the machine. This may especially be the case with 3-year-olds: Although they possess an understanding of causal mechanisms, this ability is tenuous compared to 4- and 5-year-old children [39, 40]. It is possible that providing children with more information about the causal mechanism may improve performance in future work, as one study has shown that 3-year-olds’ causal inferences depend greatly on the context of the problem (i.e., they perform better when they are told a machine likes certain objects, presumably because this provided them with an explanation for the causal rule that they understood [41]).

These findings raise important questions for future work about children’s information use and developing ability to integrate information in decision-making. Indeed, while a strong base-rate preference at the group level is clear from the current studies, the extent to which children over rely on this information (and, in turn, under use reliability information) is unclear. What’s more, 5- and 6-year-old children chose the accurate machine in Experiment 3 when base-rates were neutral, suggesting that they are not completely discrediting the reliability information. Future work using a modified procedure, in which children provide more nuanced ratings for each comparison using a graded scale, would allow us to examine flexibility in children’s ability to weigh both pieces of information. In the current experiments, we asked participants to choose one of the two machines, which has the advantages of being appropriate for testing quite young children (3- and 4-year-olds) and for its ecological validity when studying decision-making. However, it may have also masked nuances in older children’s use of base-rate and reliability information. Using a graded dependent measure would provide much needed insight on children’s ability to weigh base-rate and reliability information. Findings from the expected value literature suggest that this may be a worthwhile endeavor, as use of a scaled dependent measure has revealed that 5-year-old children vary their estimates as a function of likelihood and reward value [27, 28].

These findings also raise important questions about 4-year-old children’s ability to reconcile information. Although they were able to flexibly use base-rates and reliability information in Experiment 1, they struggled with making the correct decision when both pieces of information aligned in Experiment 3 (i.e., the perfectly reliable machine contained the better base-rate). This is particularly surprising considering that most children correctly identified which machine was more reliable, and that 4-year-olds also generally succeed in other tasks when given two pieces of information that align [19, 26]. Though we suspect that 4-year-olds’ chance performance on this easy problem may have been a sampling fluke, it is possible that our paradigm was too challenging for them to follow. Because it may be challenging for young children to consider multiple pieces of information across two machines, it would be beneficial to present children with each machine individually and ask them to provide estimates of how “good” a game is using a graded scale.

## Conclusion

In four experiments, we examined 3- to 6-year-old children’s ability to consider base-rate and reliability information in tandem when making decisions about probabilistically reliable machines. We found a strong base-rate preference in young children, which strengthened with age in some of our problems. As much prior work has examined children’s ability to reason with information in isolation, these findings raise important questions regarding the early emergence of intuitive base-rate responding, and perhaps overuse, in decision-making.

## Supporting information

S1 File Information on how the “correct” response was calculated for each problem. (PDF)
