# Supplementary material for: Young children’s use of probabilistic reliability and base-rates in decision-making
Source: PLoS One. 2022 May 25;17(5):e0268790. doi: 10.1371/journal.pone.0268790 (PMC9132303; doi:10.1371/journal.pone.0268790)
Supplement: S1 File — (PDF) [file pone.0268790.s001.pdf]

Supporting Information

For all experiments, we calculated the “correct” response by multiplying the base-rate of target objects with the reliability of each machine. We did not expect children to make these calculations explicitly.

Experiment 1

In the **100 Reliable** 100 Unreliable problem, the reliable machine gave children an 83.3% chance at a target item, while the unreliable machine gave them a 16.6% chance. In the 0 Reliable **100 Unreliable** problem, the reliable machine gave children a 0% chance, while the unreliable machine gave them a 16.6% chance.

Experiments 2a and 2b

In the 10 Reliable **90 Unreliable** problems, the reliable machine gave children an 8.3% chance at a target item, while the unreliable machine gave them a 14.4% chance. In the **30 Reliable** 70 Unreliable problems, the reliable machine gave them a 24.4% chance, while the unreliable machine gave them a 11.2% chance.

Experiment 3

In the 10 Reliable **90 Unreliable** problem, the reliable machine gave children an 10% chance at a target item, while the unreliable machine gave them a 29.7%. In the **90 Reliable** 40 Unreliable problem, the reliable machine gave children a 90% chance, while the unreliable machine gave them a 13.2% chance. In the 25 Reliable **75 Unreliable** problem, the reliable machine gave children a 25% chance, while the unreliable machine also gave them a 24.7% chance. In the **50 Reliable** 50 Unreliable problem, the reliable machine gave children a 50% chance, while the unreliable machine gave them a 16.5% chance.
